# Performance of the HEART Score in pre-hospital settings for suspected non-ST-elevation acute coronary syndrome: The POPular HEART Study

**DOI:** 10.1007/s12471-026-02041-x

**Published:** 2026-04-13

**Authors:** Jaouad Azzahhafi, Dean R. P. P. Chan Pin Yin, Mirjam Epping, Hajar Bofarid, Thijs Verhagen, Rene Boomars, Anja Radstok, Jaco Houtgraaf, Gerardus P. J. van Hout, Angela Bikker, Jurriën M. ten Berg

**Affiliations:** 1 Department of Cardiology, St. Antonius Hospital, Nieuwegein, The Netherlands; 2 Department of Cardiology, Diakonessenhuis Hospital, Utrecht, The Netherlands; 3 Emergency Medical Service Utrecht, Utrecht, The Netherlands; 4 Department of Clinical Chemistry, St. Antonius Hospital, Nieuwegein, The Netherlands; 5 Department of Cardiology, University Medical Centre Maastricht, Maastricht, The Netherlands

**Keywords:** Acute coronary syndrome, Myocardial infarction, Troponin, Point-of-care, Emergency medical services, And risk-assessment

## Abstract

**Background:**

The HEART (history, ECG, age, risk factors, and troponin) score is used to stratify patients with chest pain into low- or higher-risk for major adverse cardiac events (MACE). We assessed the diagnostic performance and interobserver agreement of the pre-hospital HEART score for ruling out myocardial infarction (MI) and MACE.

**Methods:**

This prospective, multicentre study included 383 patients with suspected non-ST-elevation acute coronary syndrome. Patients with both a pre-hospital and in-hospital HEART scores were analysed (*n* = 331). Prehospital HEART scores (based on point-of-care troponin) were assessed by ambulance personnel, and in-hospital HEART scores (based on the European Society of Cardiology 0/1-hour high-sensitivity troponin algorithm) were assessed by emergency physicians blinded to the pre-hospital scores. Endpoints were interobserver agreement (intraclass correlation coefficient, ICC) and diagnostic performance for ruling out MI and MACE at 30 days.

**Results:**

Among the 331 patients (mean age: 65 years, 48% women), 26% were classified as low risk (pre-hospital HEART ≤ 3) of whom 4.7% had an index-admission NSTEMI. Of the patients with HEART score > 3, 12.1% experienced MACE. Interobserver agreement between the pre- and in-hospital HEART scores was moderate (ICC, 0.653), with the lowest concordance for history and ECG. The pre-hospital HEART score yielded a negative predictive value of 95.33% and a sensitivity of 91.7% for MACE at 30 days.

**Conclusion:**

Pre- and in-hospital HEART scores showed moderate agreement. The 30-day MACE rate (4.7%) in the pre-hospital low-risk group indicates that improved training in history and ECG assessment, and use of high-sensitivity assays are required.

**Supplementary Information:**

The online version of this article (10.1007/s12471-026-02041-x) contains supplementary material, which is available to authorized users.

## What’s new?

- A modified pre‑hospital HEART score using point‑of‑care cTnI was prospectively applied by ambulance personnel and compared with in‑hospital ESC 0/1‑h hs‑troponin algorithms.
- Despite 26% being classified as low risk, the 30‑day MACE rate was 4.7%, exceeding the 2% safety threshold and indicating limited safety of pre‑hospital HEART‑score use without hs‑troponin assays.
- In‑hospital reassessment reduced the low‑risk event rate to 1.7%, and diagnostic safety improved further in patients with ≥2 hours of symptom.

## Introduction

Chest pain accounts for approximately 10% of all emergency department (ED) presentations worldwide [1, 2]. Acute myocardial infarction remains a critical. [3] While ST-elevation myocardial infarction (STEMI) enables rapid triage, non-ST-elevation acute coronary syndrome (NSTE-ACS) poses a diagnostic challenge because ECG changes may be subtle, and troponin requires time to rise [4].

Patients suspected of NSTE-ACS are frequently transferred despite a low probability of NSTE-ACS, placing a burden on healthcare and underscoring the need for prehospital risk stratification to identify low-risk patients earlier [5, 6].

The applicability of established NSTE-ACS risk tools to unselected pre-hospital chest pain patients is limited [6–8]. Previous pre-hospital studies have focused on adaptations of the HEART (history, ECG, age, risk factors, and troponin) score combined with point-of-care (POC)-troponin, mostly in single-centre cohorts or relied on pre-hospital use of high-sensitivity assays not yet routinely available and without using the formal in-hospital European Society of Cardiology (ESC) 0/1-hour high-sensitivity troponin (hs-cTn) algorithm as the diagnostic reference [9]. The HEART score is used to stratify patients into low- or higher-risk groups for major adverse cardiac events (MACE) at 30 days [8, 10]. While its utility in ED settings has been widely demonstrated, evidence on its prehospital use is limited, partly because of the logistical burden of off-site troponin testing [11].

Pre-hospital risk assessment could relieve ED crowding by keeping truly low-risk patients at home and directing high-risk patients to definitive care (e.g., invasive diagnostics and/or coronary revascularisation) [12, 13]. However, its real-world value remains uncertain, as most ambulance services still lack access to POC hs-cTn assays and the current evidence is largely single-centre [14].

We assessed diagnostic performance and interobserver agreement between prehospital and in-hospital HEART scores in ruling out myocardial infarction (MI) and MACE at 30 days in suspected NSTE-ACS patients. To our knowledge, this is the first multicentre prehospital study to directly compare the HEART score assessment, based on POC conventional troponin I, with the in-hospital ESC 0/1-hour hs-cTn algorithm as the diagnostic reference.

## Methods

### Study design

**Fig. 1:**
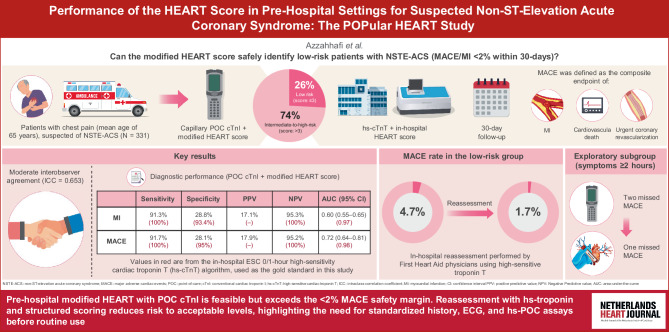
Infographic: Performance of the HEART Score in Pre-Hospital Settings for Suspected Non-ST-Elevation Acute Coronary Syndrome

The POPULAR HEART study is an investigator-initiated prospective multicentre observational study evaluating diagnostic accuracy and interobserver agreement of pre-hospital HEART score assessment combined with POC troponin testing in suspected NSTE-ACS patients, see Fig. 1.

### Study population

Adults (≥ 18 years) with chest pain suspected of having NSTE-ACS transported by ambulance were eligible. Exclusion criteria are shown in the Electronic Supplementary Material [ESM] Box S1. Only patients with both pre-hospital and in-hospital HEART scores and complete 30-day follow-up were included in the complete-case analysis. Additional methodological details are provided in ESM Box S1.

### Study protocol

Ambulance personnel calculated the pre-hospital HEART score by using a POC conventional cardiac troponin I (cTnI) assay and capillary blood samplingIn-hospital HEART score were calculated by physicians blinded to pre-hospital results using the in-hospital high-sensitivity (hs)-cTnT, (ESM Box S2). All clinicians received standardized briefing atinitiation regarding HEART score assessment and interpretation of its individual components. In-hospital HEART scores were subsequently assessed by different physicians as part of routine clinical care.

### Troponin testing and cutoff values

Troponin cutoff followedmanufacturer guidance and ESC recommendations (ESM Box S3). Prior to study roll-out, capillary whole blood use on the i‑STAT platform was locally verified for the intended application.

### Primary analysis

We compared diagnostic performance of pre-hospital HEART scoring using POC conventional cTnI testing versus the in-hospital ESC 0/1 h rule-out algorithm using hs-cTnT to identify low-risk patients with suspected NSTE-ACS.

The pre-hospital HEART score was assessed at first medical contact (T0) by ambulance personnel using capillary blood and a POC conventional cTnI assay (i‑STAT 1, Abbott Point of Care Inc., Princeton, NJ, USA). The reference standard consisted of serial venous hs‑cTnT measurements obtained upon hospital arrival (T1) and, when clinically indicated, repeated 1 h later (T2) and interpreted in accordance with the ESC 0/1‑h rule-out algorithm.

### Exploratory sub-analyses

**Fig. 2:**
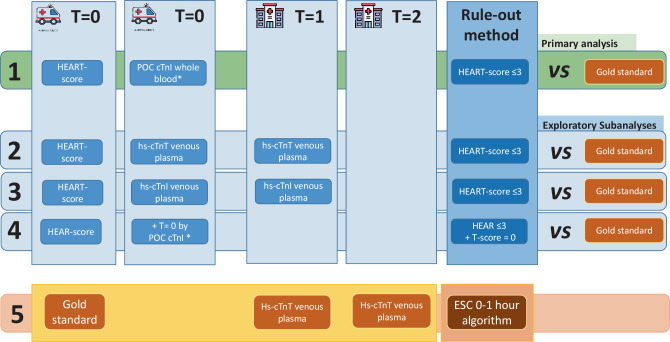
Schematic of the four diagnostic strategies compared with the in-hospital reference standard. All T0 samples were obtained during the first medical contact in the ambulance. T1 refers to blood drawn upon hospital arrival, and T2 to the 1‑hour follow-up sample. The reference standard consisted of serial in-hospital high-sensitive cardiac troponin T (hs-cTnT) measurements, interpreted according to the ESC 0/1-hour algorithm. 1. Prehospital HEART score using point-of-care (POC) conventional cardiac troponine I (cTnI) (primary strategy): HEART score was calculated by paramedics at T0 using capillary blood samples and POC conventional cTnI assay (i-STAT, Abbott). 2. Alternate prehospital HEART score incorporating serial hs-cTnT and hs-cTnI scores (central laboratory): HEART score calculated using hs-cTnT and hs-cTnI values at T0 and T1, with the troponin component scored 0, 1, or 2 points based on the ESC 0/1-hour algorithm (rule-out, observation, and rule-in). 3. Combined pre-hospital HEAR ≤ 3 and POC conventional cTnI score = 0 strategy: Patients were classified as low risk only when both the HEAR score was ≤ 3 and the troponin sub-score (from strategy 1) was 0. 4. Reference standard (gold standard): Serial in-hospital hs-cTnT measurements at T1 and T2 interpreted using the ESC 0/1-hour algorithm to confirm or exclude myocardial infarction based on dynamic changes

Details of exploratory sub-analyses are provided in ESM Box S1 and Fig. 2.

### Endpoints

Endpoints were interobserver agreement between the pre- and in-hospital HEART scores and diagnostic performance of prehospital HEART scores in ruling out 30-day MI and MACE (ESM Box S1).

### Statistical analysis

Interobserver agreement was assessed using ICCs, interpreted as follows: < 0.50 (poor), 0.50–0.75 (moderate), 0.75–0.90 (good), and > 0.90 (excellent) [11]. The diagnostic accuracy was determined based on sensitivity, specificity, positive predictive value (PPV), and negative predictive value (NPV) (ESM Box S1).

### Power calculation

For the inter-observer agreement analysis, 145 paired HEART-score assessments were required (target ICC > 0.80 α = 0.025, power = 80%). In all, 331 paired assessments were conducted. For diagnostic performance analyses, the sample size was sufficient based on expected event rates. See ESM Box S1 for detailed power calculation, non-inferiority comparisons, recruitment, and follow-up.

### Ethical considerations

This study adhered to the Declaration of Helsinki, was approved by the institutional Medical Research Ethics Committee, and all patients provided written informed consent.

## Results

### Study population

**Fig. 3:**
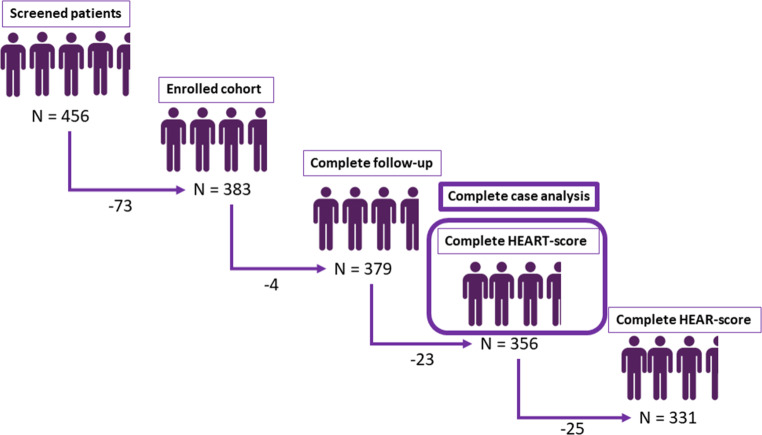
Flowchart of patient inclusion and analytical cohorts

Between August 2020 and August 2023, 456 patients with suspected NSTE-ACS were screened. Of them, 383 (84%) consented and 379 (99%) completed follow-up, and pre- and in-hospital HEART scores were available for 331 patients (86.4%), see Fig. 3.

### Baseline characteristics

The mean age of the 331 complete patients was 65.4 years; 48.0% were female. Cardiovascular risk factors were commonly observed; 28.0% had prior MIBased on pre-hospital HEART-scores 26.0% were low risk(*n* = 86) and 74.0% intermediate-to-high-risk (*n* = 245; Tab. 1). The 30-day MACE rate was 4.7% in the low-risk group (all index NSTEMIs) and 12.1% in the higher-risk group.

**Table 1:** Baseline characteristics based on pre-hospital HEART score

Characteristics	All (*n* = 331)	HEART ≤ 3 (*n* = 86)	HEART > 3 (*n* = 245)	*p*-value
Mean age, years (SD)	65.43 (13.51)	56.08 (13.48)	68.71 (11.92)	< 0.001
Female, *n* (%)	159 (48.0)	53 (61.6)	106 (43.3)	0.005
Diabetes mellitus, *n* (%)	73 (22.1)	11 (12.8)	62 (25.3)	0.024
Body mass index ≥ 30 kg/m^2^, *n* (%)	94 (28.5)	28 (32.6)	66 (27.0)	0.404
Hypercholesterolemia, *n* (%)	175 (53.0)	26 (30.2)	149 (61.1)	< 0.001
Hypertension, *n* (%)	182 (55.0)	21 (24.4)	161 (65.7)	< 0.001
Positive family history, *n* (%)	114 (34.4)	29 (33.7)	85 (34.7)	0.975
Current smoker, *n* (%)	54 (16.3)	15 (17.4)	39 (15.9)	0.873
Previous myocardial infarction, *n* (%)	92 (28.0)	4 (4.7)	88 (36.2)	< 0.001
Previous PCI, *n* (%)	82 (24.9)	4 (4.7)	78 (32.1)	< 0.001
Previous CABG, *n* (%)	30 (9.1)	0 (0.0)	30 (12.3)	0.001
Previous TIA/stroke, *n* (%)	25 (7.6)	1 (1.2)	24 (9.8)	0.018
Previous peripheral artery disease, *n* (%)	7 (2.1)	0 (0.0)	7 (2.9)	0.251
Previous heart failure, *n* (%)	12 (3.6)	0 (0.0)	12 (4.9)	0.079
Previous chronic kidney disease, *n* (%)	4 (1.2)	0 (0.0)	4 (1.6)	0.536
Active malignancy, *n* (%)	14 (4.2)	4 (4.7)	10 (4.1)	1.000
COPD, *n* (%)	25 (7.6)	2 (2.3)	23 (9.4)	0.058
Atrial fibrillation, *n* (%)	46 (13.9)	3 (3.5)	43 (17.6)	0.002

*PCI* percutaneous coronary intervention, *CABG* coronary artery bypass grafting, *TIA* transient ischaemic attack, *COPD* chronic obstructive pulmonary disease

### Agreement between pre-hospital and in-hospital HEART scores

In-hospital assessment reclassified 35 patients to low-risk (*n* = 121/331, 36.6%) (ESM Table S1). Outcomes in the pre-hospital low-risk group are summarised in ESM Table S2. The overall concordance between pre- and in-hospital scores was moderate (ICC = 0.653, with lowest concordance for history and ECG; ESM Table S3). Differences between pre-hospital and in-hospital HEART risk classification are summarised in ESM Table S4 and Table S5, showing concordant low-risk classification in 66 patients and discordant classification in 75 patients. Outcomes in the false-negative subgroup are detailed in ESM Table S6. Among pre-hospital low-risk patients (*n* = 86), 66 (76.7%) remained low risk after in-hospital assessment. Interobserver agreement for the total HEART score within this pre-hospital low-risk subgroup was low (ICC 0.16). Component-level interobserver agreement within the pre-hospital low-risk group is provided in ESM Table S3a. A sensitivity analysis correcting the age component based on actual age at inclusion for both pre-hospital and in-hospital assessments resulted in only minimal changes in overall agreement (ICC 0.653 to 0.668) and led to reclassification of four patients (1.2%), all shifting from a HEART score of 3 to 4 (i.e., from low-risk to intermediate-risk). None of whom experienced a MACE during follow-up (ESM Table S3b).

### *Diagnostic accuracy in predicting MI and MACE at 30 days follow-up*

The diagnostic performance of the HEART score in predicting MI and MACEs within 30 days is summarised in Tab. 2 and 3.

**Table 2:** Diagnostic accuracy of prehospital strategies for diagnosing myocardial infarction

Diagnostic strategy	Sensitivity (%)	Specificity (%)	PPV (%)	NPV (%)	AUC (95% CI)
*Primary analysis*					
Pre-hospital HEART ≤ 3 with POC conventional cTnI	91.3	28.8	17.1	95.3	0.60 (0.55–0.65)^a^
*Exploratory sub-analyses*					
Pre-hospital HEAR ≤ 3 + T0 POC conventional cTnI = 0	93.5	28.4	17.4	96.4	0.39 (0.35–0.44)^a^
Pre-hospital HEART ≤ 3 with serial hs-cTnT	95.1	12.9	14.6	94.4	0.70 (0.62–0.79)^b^
Pre-hospital HEART ≤ 3 with serial hs-cTnI	89.7	26.8	15.5	94.5	0.75 (0.65–0.85)^c^
*Golden standard: hs-cTn assays*					
In-hospital ESC 0/1‑h hs-cTnT reference (T1–T2)^d^	100	93.4	80.5	100	0.97 (0.95–0.99)

^a^*p*-value < 0.001 compared to the golden standard

^b^*P*-value 0.06

^c^*P*-value = 0.27

^d^Golden standard = ESC 0‑/1-hour in-hospital algorithm high-sensitive Troponin T

*POC* point of care, *cTnI* cardiac troponin I, *hs-cTnI* high-sensitive cardiac troponin I, *hs-cTnT* high-sensitive cardiac troponin T

**Table 3:** Diagnostic accuracy of prehospital strategies using the HEART score for diagnosing major adverse cardiac events

Diagnostic strategy	Sensitivity (%)	Specificity (%)	PPV (%)	NPV (%)	AUC (95% CI)
*Primary analysis*					
Pre-hospital HEART ≤ 3 with POC conventional cTnI	91.7	28.1	17.9	95.2	0.72 (0.64–0.81)^a^
*Exploratory sub-analyses*					
Pre-hospital HEAR ≤ 3 + T0 POC conventional cTnI = 0	94.4	27.6	18.3	96.7	0.39 (0.34–0.44)^a^
Pre-hospital HEART ≤ 3 with serial hs-cTnT	95.3	13.0	15.3	94.4	0.70 (0.62–0.79)^b^
Pre-hospital HEART ≤ 3 with serial hs-cTnI	90.3	27.1	16.7	94.5	0.75 (0.66–0.85)^c^
*Golden standard: hs-cTn assays*					
In-hospital ESC 0/1‑h hs-cTnT reference (T1–T2) ^d^	100	95	87.1	100	0.98 (0.95–0.99)

^a^*p-*value < 0.001 compared to the golden standard

^b^*p-*value = 0.054

^c^*P-*value = 0.21

^d^Golden standard = ESC 0‑/1-hour in-hospital algorithm high-sensitive Troponin T

*POC* point of care, *cTnI* cardiac troponin I, *hs-cTnI* high-sensitive cardiac troponin I, *hs-cTnT* high-sensitive cardiac troponin T

### Primary analysis

The pre-hospital HEART score of ≤ 3 obtained in the ambulance with POC conventional cTnI yielded a sensitivity of 91.3% and specificity of 28.8% for MI (NPV 95.3%, area under the curve (AUC) of 0.60 (95% confidence interval [CI]: 0.55–0.65). For predicting MACE, the sensitivity was 91.7%, the specificity was 28.1% (NPV 95.2%, AUC 0.72 [95% CI: 0.64–0.81]). The in-hospital ESC 0/1‑h hs-cTnT algorithm (reference standard) achieved 100% sensitivity and NPV for MI and MACE (specificity was 93.4% and 95, and AUC was 0.97 and 0.98, respectively).

### Exploratory sub-analyses

Alternative strategies, including pre-hospital HEART scores incorporating serial hs-cTnT and hs-cTnI measurements, or the use of a stricter definition requiring HEAR ≤ 3 and troponin sub-score of 0, showed higher sensitivity but at the cost of reduced specificity (ESM Box S1; Tab. 2 and 3, clinical endpoints are described in ESM Tables S10, S11 and S15).

### Clinical outcomes in the pre-hospital low-risk group

Among the 86 pre-hospital low-risk patients (26% of the cohort), four MACEs occurred (4.7%); all were index-admission NSTEMIs, with no urgent revascularization or cardiovascular mortality during follow-up (ESM Tables S2 and S7). After in-hospital reassessment by Cardiac Emergency physicians, 35 patients were reclassified as low-risk, yielding a total of 121 patients. In this extended low-risk group, only two experienced MACEs (1.7%); both patients were already identified as low-risk at the pre-hospital assessment stage (ESM Table S8).

### Influence of symptom duration

Among 331 patients, 193 (58%) had symptoms lasting ≥ 2 h (2 h). Based on the pre-hospital HEART score, 46 patients (23.8%) were low risk, and two (4.3%) experienced MACE (ESM Table S12). Further duration-based hs-troponin analyses are shown in ESM Tables S13 (hs-cTnT) and Table S14 (hs-cTnI), with overall diagnostic performance in ESM Table S15.

### Discharge diagnoses

ACS was confirmed in 62/331 participants (18.7%). Four events occurred in the pre-hospital low-risk group (4.7%, all NSTEMIs), whereas the remaining 58 occurred in patients classified as intermediate-to-high (23.7%).

Benign thoracic pain was the most common discharge diagnosis (121/331, 36.6%), followed by non-ischaemic cardiac conditions (52/331, 15.7%) and non-cardiac disorders (92/331, 27.8%), with the latter occurring nearly twice as often in the pre-hospital low-risk cohort (43.0% vs. 22.0%), see ESM Table S9). Additional subgroup details are provided in ESM Box S4.

## Discussion

This prospective multicentre study demonstrated moderate agreement between the pre- and in-hospital HEART scores. While approximately one in four patients was classified as low-risk based on the pre-hospital HEART score, the observed MACE rate of 4.7% in this group exceeded the 2% safety threshold often used to justify early discharge decisions. The prehospital HEART score showed high sensitivity and NPV but only modest accuracy in predicting MACE at 30 days compared with the in-hospital algorithm, indicating the need for further optimisation (e.g., structured training and potentially the use of hs-assays).

Two of the four missed MACE could have been prevented if subjective components (history, ECG) had been scored differently or if high-sensitivity assays had been used. Age misclassification did not contribute to missed events and was mainly attributable to incorrect scoring, as correction for actual age had a negligible impact on agreement or clinical outcomes. Although all ambulance personnel and Cardiac Emergency physicians received standardized training at study initiation, HEART scores were calculated by different clinicians during routine care rather than by a dedicated study team. In addition, Cardiac Emergency physicians may have had access to more extensive clinical information, which could partly explain the lower agreement observed for subjective components such as history. This contrasts with retrospective in-hospital studies using structured chart abstraction and consensus scoring, which report higher interobserver agreement and no missed MACEs in low-risk patients [15, 16]. In this context, in-hospital reassessment using hs-cTnT instead of conventional POC troponin reduced the event rate in the low-risk group to 1.7%, approaching the commonly accepted safety margin. Boeddinghaus et al. showed that the use of an hs-cTnI device combined with a 0/1 h algorithm reached 100% NPV for ruling out MI [3, 14]. Although our exploratory use of serial hs-cTn in the pre-hospital HEART score was based on retrospective central laboratory high-sensitivity troponin test results, it did reclassify two of the four missed MACEs.

Our findings suggest that incorporating symptom duration, including serial hs-Tn testing, into the pre-hospital HEART score can enhance diagnostic safety. In patients with symptom onset ≥ 2 h, applying the pre-hospital HEART score reduced missed MACEs from two to one, while retaining a comparable low-risk proportion [9, 17–19].

### Clinical implications

This study demonstrated that pre-hospital use of the HEART score can safely identify a proportion (26%) of patients with suspected NSTE-ACS as low risk, potentially avoiding hospital transport when appropriate training and safeguards are in place. However, our observed 4.7% MACE rate in the pre-hospital low-risk group exceeds the 2% safety threshold for ED discharge [20]. This benchmark is not absolute; prior studies in primary care have reported missed ACS rates of up to 2.6%, underscoring the need to contextualise safety thresholds within the pre-hospital setting [2].

Importantly, systematic training for interpreting the subjective HEART components (history, ECG findings, and risk factors) may further reduce misclassification. In our cohort, two of the four missed MACE occurred in patients who were incorrectly classified as low risk owing to the subjective underestimation of HEART components and false-negative POC conventional cTnI readings. Both were correctly reclassified after in-hospital reassessment (using hs-cTnT), suggesting that if similar improvements are applied in the pre-hospital setting through structured training and the use of a (POC) hs-cTn assay, the missed event rate could theoretically decrease from 4.7% to 1.7%.

### Limitations

Several limitations must be acknowledged. First, the intended sample size of 650 patients was not reached due to limited availability of the POC conventional cTnI device (i-STAT 1, Abbott) used in the pre-hospital setting. Second, the COVID-19 pandemic disrupted patient enrolment for 6–12 months, delaying progress and diverting the ambulance focus towards clinical care. Third, technical problems affected the completeness of the data. Finally, the study was terminated early in August 2023 because of the reduced relevance of the POC conventional cTnI assay, as high-sensitivity cTn assays became available. Despite these challenges, the study successfully met two objectives: evaluating interobserver agreement and diagnostic accuracy of pre-hospital HEART score risk stratification using POC troponin.

### Recommendations for future research

Future research should focus on optimising and carefully monitoring the implementation of pre-hospital low-risk identification through structured training and the use of high-sensitivity troponin assays. At the same time, the greatest potential healthcare impact is likely to be achieved by safely refining pre-hospital pathways for the substantially larger intermediate-risk population (HEART score 4–6), particularly in patients with negative or minimally elevated hs-cTn levels. In addition, defining the optimal symptom duration thresholds for pre-hospital application of both biomarker-based and HEART score-based strategies, including the role of repeat sampling in routine ambulance care, will be essential to further improve safety and efficiency while reducing ED burden.

## Conclusion

This prospective multicentre study showed that the agreement between pre- and in-hospital HEART score assessments was moderate. The pre-hospital HEART score classified 26% of patients as low risk; the 30-day MACE rate of 4.7%, exceeding the 2% safety threshold. In-hospital reassessment, including the assessment of hs-cTnT levels, reduced the low-risk event rate to 1.7%. Safety can be improved through paramedic training, history, ECG assessment, and hs-cTn assays. These results support the cautious and stepwise implementation of routine care.

## Supplementary Information

ESM1: Supplementary material 1
