# Supplementary material for: Performance of the HEART Score in pre-hospital settings for suspected non-ST-elevation acute coronary syndrome: The POPular HEART Study
Source: Neth Heart J. 2026 Apr 13;34(5):179–88. doi: 10.1007/s12471-026-02041-x (PMC13090458; doi:10.1007/s12471-026-02041-x)
Supplement: Supplementary file 1 — ESM1: Supplementary material 1 [file 12471_2026_2041_MOESM1_ESM.docx]

**Performance of the HEART Score in Pre-hospital Settings for Suspected Non-ST-Elevation Acute Coronary Syndrome: The POPular HEART Study**

**Supplementary Table 1.** Baseline in-hospital HEART scores

| Characteristics | All (n = 331) | HEART ≤ 3 (n = 121) | HEART > 3 (n = 210) | p-value |
| --- | --- | --- | --- | --- |
| Mean age, years (SD) | 65.43 (13.51) | 57.78 (13.16) | 69.83 (11.63) | <0.001 |
| Female, n (%) | 159 (52.0) | 73 (60.3) | 86 (41.0) | 0.001 |
| Diabetes mellitus, n (%) | 73 (22.1) | 14 (11.6) | 59 (28.1) | 0.001 |
| Body mass index ≥ 30 kg/m2, n (%) | 94 (28.7) | 40 (33.1) | 54 (26.1) | 0.222 |
| Hypercholesterolemia, n (%) | 175 (53.0) | 36 (29.8) | 139 (66.5) | <0.001 |
| Hypertension, n (%) | 182 (55.0) | 32 (26.4) | 150 (71.4) | <0.001 |
| Positive family history, n (%) | 114 (34.4) | 36 (29.8) | 78 (37.1) | 0.214 |
| Current smoker, n (%) | 54 (16.3) | 19 (15.7) | 35 (16.7) | 0.941 |
| Previous myocardial infarction, n (%) | 92 (28.0) | 6 (5.0) | 86 (41.5) | <0.001 |
| Previous PCI, n (%) | 82 (24.9) | 3 (2.5) | 79 (38.0) | <0.001 |
| Previous CABG, n (%) | 30 (9.1) | 1 (0.8) | 29 (13.9) | <0.001 |
| Previous TIA/stroke, n (%) | 25 (7.6) | 4 (3.3) | 21 (10.0) | 0.045 |
| Previous peripheral artery disease, n (%) | 7 (2.1) | 1 (0.8) | 6 (2.9) | 0.401 |
| Previous heart failure, n (%) | 12 (3.6) | 1 (0.8) | 11 (5.2) | 0.078 |
| Previous chronic kidney disease, n (%) | 4 (1.2) | 1 (0.8) | 3 (1.4) | 1.000 |
| Active malignancy, n (%) | 14 (4.2) | 2 (1.7) | 12 (5.7) | 0.138 |
| COPD, n (%) | 25 (7.6) | 3 (2.5) | 22 (10.5) | 0.015 |
| Atrial fibrillation, n (%) | 46 (13.9) | 9 (7.4) | 37 (17.6) | 0.016 |

**Supplementary Table 2.** Cases of NSTEMI in the low-risk pre-hospital group (HEART-score ≤3)

| Study ID | Ambu Total | EHH Total | ΔH | ΔE | ΔA | ΔR | ΔT | NSTEMI | PCI Received | CABG Received |
| --- | --- | --- | --- | --- | --- | --- | --- | --- | --- | --- |
| PH-AZN-178 | 2 | 8 | +1 | +1 | 0 | +2 | +2 | Yes | No | No |
| PH-AZN-355 | 3 | 3 | 0 | +1 | 0 | 0 | -1 | Yes | No | No |
| PH-AZN-774 | 3 | 5 | +1 | 0 | 0 | 0 | +1 | Yes | No | No |
| PH-AZN-174 | 1 | 3 | +2 | 0 | 0 | 0 | 0 | Yes | No | No |

**Supplementary Table 3.** Intraclass Correlation Coefficient (ICC) of agreement between pre- and in-hospital HEART scores

| HEART-score Component | ICC | Agreement (%) |  |
| --- | --- | --- | --- |
| History | 0.212 | 42.9 |  |
| ECG | 0.387 | 67.1 |  |
| Age | 0.905 | 94.9 |  |
| Risk Factors | 0.542 | 62.2 |  |
| Troponin | 0.46 | 69.8 |  |
| Total HEART-score | 0.653 | 27.2 |  |

**Supplementary Table 3a.** Interobserver agreement for the individual HEART score components and the total HEART score was assessed in patients classified as low risk in the pre-hospital setting (HEART ≤3; n=86). Agreement was quantified using a two-way random-effects intraclass correlation coefficient with absolute agreement. While agreement for age was excellent, agreement for subjective components, particularly history, was low. The overall interobserver agreement for the total HEART score in this subgroup was low (ICC 0.155).

| HEART-score Component | ICC | Agreement (%) |  |
| --- | --- | --- | --- |
| History | -0.074 | 41.9 |  |
| ECG | 0.388 | 81.4 |  |
| Age | 0.954 | 96.5 |  |
| Risk Factors | 0.342 | 62.8 |  |
| Troponin | 0.204 | 88.4 |  |
| Total HEART-score | 0.155 | 27.9 |  |

**Supplementary Table 3b. Clinical impact of age misclassification on low-risk classification**

| Setting | Total (n) | False high* n (%) | False low† n (%) | MACE in reclassified patients |
| --- | --- | --- | --- | --- |
| Pre-hospital | 331 | 0 (0.0) | 3 (0.9) | 0 |
| In-hospital | 331 | 1 (0.3) | 0 (0.0) | 0 |

* False high: not classified as low risk before age correction but low risk after correction
† False low: classified as low risk before age correction but not after correction

**Supplementary Table 4.** Agreement between pre- and in-hospital HEART score classifications in the total cohort of 331 patients

| Classification | No. of patients |
| --- | --- |
| True Negatives (pre-hospital ≤3 & in-hospital ≤3) | 66 |
| False Negatives (pre-hospital ≤3 but in-hospital >3) | 20 |
| False Positives (pre-hospital >3 but in-hospital ≤3) | 55 |
| True Positives (pre-hospital >3 & in-hospital >3) | 190 |

**Supplementary Table 5.** Differences in HEART score components

| Component | Total  Similar | Total Lower | Total Higher | False Negative^1^  Similar | False Negative Lower | False Negative Higher | False Positive^2^  Similar | False Positive Lower | False Positive Higher |
| --- | --- | --- | --- | --- | --- | --- | --- | --- | --- |
| History, n (%) | 142 (12.7%) | 37 (14.1%) | 152 (54.7%) | 9 (14.1%) | 11 (31.4%) | 0 (0%) | 2 (1.1%) | 0 (0%) | 53 (62.4%) |
| ECG, n (%) | 222 (19.9%) | 58 (22.1%) | 51 (18.3%) | 13 (20.3%) | 7 (20%) | 0 (0%) | 46 (24.6%) | 1 (33.3%) | 8 (9.4%) |
| Age, n (%) | 314 (28.2%) | 12 (4.6%) | 5 (1.8%) | 18 (28.1%) | 2 (5.7%) | 0 (0%) | 53 (28.3%) | 0 (0%) | 2 (2.4%) |
| Risk Factors, n (%) | 206 (18.5%) | 74 (28.2%) | 51 (18.3%) | 9 (14.1%) | 10 (28.6%) | 1 (100%) | 35 (18.7%) | 1 (33.3%) | 19 (22.4%) |
| Troponin, n (%) | 231 (20.7%) | 81 (30.9%) | 19 (6.8%) | 15 (23.4%) | 5 (14.3%) | 0 (0%) | 51 (27.3%) | 1 (33.3%) | 3 (3.5%) |

¹ False negatives = Pre-hospital HEART score ≤3, but in-hospital HEART score >3. This indicates that the ambulance classified a patient as low risk, whereas the hospital classified the same patient as high risk.
² False positives = Pre-hospital HEART score >3, but in-hospital HEART score ≤3. This indicates that the ambulance classified a patient as high-risk, whereas the hospital classified the same patient as having a lower risk.

**Supplementary Table 6.** Primary endpoints during 30-days follow-up for false negatives. False negatives were defined as patients with a prehospital HEART score of <3 but an in-hospital HEART score of >3.

| Outcome | False-Negatives (n, %) |
| --- | --- |
| Total Patients | 20 (100%) |
| MACE | 2 (10.0%) |
| CV Deaths | 0 (0.0%) |
| Non-CV Deaths | 0 (0.0%) |
| ACS | 2 (10.0%) |
| STEMI | 0 (0.0%) |
| NSTEMI | 2 (10.0%) |
| Unstable Angina Pectoris | 0 (0.0%) |
| Type 2 MI | 0 (0.0%) |
| Other non-ACS cardiac condition | 3 (15.0%) |
| PCI | 0 (0.0%) |
| Urgent PCI | 0 (0.0%) |
| CABG | 0 (0.0%) |
| Urgent CABG | 0 (0.0%) |
| CVA/TIA | 0 (0.0%) |
| TIA | 0 (0.0%) |
| Readmission | 0 (0.0%) |
| Cardiac Readmission | 0 (0.0%) |
| Non-Cardiac Readmission | 0 (0.0%) |

*MACE = Cardiovascular mortality, myocardial infarction (STEMI/NSTEMI), urgent revascularization at 30 days; ACS = Acute Coronary Syndrome, including STEMI/NSTEMI/UAP; PCI = Percutaneous Coronary Intervention; CABG = Coronary Artery Bypass Grafting; CVA/TIA = Cerebrovascular accident or transient ischemic attack*

**Supplementary Table 7.** Primary endpoints during the 30-days follow-up in the prehospital group (based on the prehospital HEART score)

| Clinical endpoints | Total (n = 331) | HEART-score ≤3 (n = 86) | HEART-score >3 (n = 245) |
| --- | --- | --- | --- |
| MACE | 46 (13.9%) | 4 (4.7%) | 42 (17.1%) |
| All-cause mortality | 2 (0.6%) | 0 (0%) | 2 (0.8%) |
| Cardiovascular mortality | 2 (0.6%) | 0 (0%) | 2 (0.8%) |
| Non-CV mortality | 0 (0%) | 0 (0%) | 0 (0%) |
| ACS | 62 (18.7%) | 4 (4.7%) | 58 (23.7%) |
| STEMI | 8 (2.4%) | 0 (0%) | 8 (3.3%) |
| NSTEMI | 37 (11.2%) | 4 (4.7%) | 33 (13.5%) |
| Unstable angina pectoris | 16 (4.8%) | 0 (0%) | 16 (6.5%) |
| Type 2 MI | 4 (1.2%) | 1 (1.2%) | 3 (1.2%) |
| Other cardiac condition (non-ACS) | 52 (15.7%) | 11 (12.8%) | 41 (16.7%) |
| PCI | 3 (0.9%) | 0 (0%) | 3 (1.2%) |
| Urgent PCI | 2 (0.6%) | 0 (0%) | 2 (0.8%) |
| CABG | 1 (0.3%) | 0 (0%) | 1 (0.4%) |
| Urgent CABG | 1 (0.3%) | 0 (0%) | 1 (0.4%) |
| CVA | 1 (0.3%) | 0 (0%) | 1 (0.4%) |
| TIA | 0 (0%) | 0 (0%) | 0 (0%) |
| Admission | 77 (23.3%) | 7 (8.1%) | 70 (28.6%) |
| Readmission | 20 (6%) | 3 (3.5%) | 17 (6.9%) |
| Cardiac readmission | 14 (4.2%) | 1 (1.2%) | 13 (5.3%) |
| Non-cardiac readmission | 6 (1.8%) | 2 (2.3%) | 4 (1.6%) |

*MACE = Cardiovascular mortality, myocardial infarction (STEMI/NSTEMI), urgent revascularization at 30 days; ACS = Acute Coronary Syndrome, including STEMI/NSTEMI/UAP; PCI = Percutaneous Coronary Intervention; CABG = Coronary Artery Bypass Grafting; CVA/TIA = Cerebrovascular accident or transient ischemic attack*

**Supplementary Table 8.** Primary endpoints during 30-days follow-up in the in-hospital group

| Clinical endpoints | Total (n = 331) | HEART-score ≤3 (n = 121) | HEART-score >3 (n = 210) |
| --- | --- | --- | --- |
| MACE | 46 (13.9%) | 2 (1.7%) | 44 (21%) |
| All-cause mortality | 2 (0.6%) | 0 (0%) | 2 (1%) |
| Cardiovascular mortality | 2 (0.6%) | 0 (0%) | 2 (1%) |
| Non-CV mortality | 0 (0%) | 0 (0%) | 0 (0%) |
| ACS | 62 (18.7%) | 3 (2.5%) | 59 (28.1%) |
| STEMI | 8 (2.4%) | 0 (0%) | 8 (3.8%) |
| NSTEMI | 37 (11.2%) | 2 (1.7%) | 35 (16.7%) |
| Unstable angina pectoris | 16 (4.8%) | 1 (0.8%) | 15 (7.1%) |
| Type 2 MI | 4 (1.2%) | 1 (0.8%) | 3 (1.4%) |
| Other cardiac condition (non-ACS) | 52 (15.7%) | 13 (10.7%) | 39 (18.6%) |
| PCI | 3 (0.9%) | 1 (0.8%) | 2 (1%) |
| Urgent PCI | 2 (0.6%) | 0 (0%) | 2 (1%) |
| CABG | 1 (0.3%) | 0 (0%) | 1 (0.5%) |
| Urgent CABG | 1 (0.3%) | 0 (0%) | 1 (0.5%) |
| CVA | 1 (0.3%) | 0 (0%) | 1 (0.5%) |
| TIA | 0 (0%) | 0 (0%) | 0 (0%) |
| Admission | 77 (23.3%) | 7 (5.8%) | 70 (33.3%) |
| Readmission | 20 (6%) | 4 (3.3%) | 16 (7.6%) |
| Cardiac readmission | 14 (4.2%) | 2 (1.7%) | 12 (5.7%) |
| Non-cardiac readmission | 6 (1.8%) | 2 (1.7%) | 4 (1.9%) |

*MACE = Cardiovascular mortality, myocardial infarction (STEMI/NSTEMI), urgent revascularization at 30 days; ACS = Acute Coronary Syndrome, including STEMI/NSTEMI/UAP; PCI = Percutaneous Coronary Intervention; CABG = Coronary Artery Bypass Grafting; CVA/TIA = Cerebrovascular accident or transient ischemic attack*

**Supplementary Table 9.** Discharge diagnosis based on pre-hospital HEART score

| Diagnosis | Total (n = 331) | HEART ≤3 (n = 86) | HEART >3 (n = 245) |
| --- | --- | --- | --- |
| Unstable Angina Pectoris | 16 (4.8%) | NA | 16 (6.5 %) |
| NSTEMI | 36 (10.9%) | 4 (4.7%) | 32 (13.1%) |
| STEMI | 8 (2.4%) | NA | 8 (3.3%) |
| Semi-recent MI | 2 (0.6%) | NA | 2 (0.8%) |
| Type 2 MI | 4 (1.2%) | 1 (1.1%) | 3 (1.2%) |
| Non-ischemic Cardiac Conditions | 52 (15.7%) | 11 (12.8%) | 41 (16.7%) |
| Other non-cardiac Condition | 92 (27.8%) | 37 (43.0%) | 55 (22.4%) |
| Thoracic Pain e.c.i. | 121 (36.6%) | 33 (38.4%) | 88 (35.9%) |

*NSTEMI = non-ST elevation myocardial infarction; STEMI = ST-elevation myocardial infarction*

**Supplementary Table 10.** Primary endpoints during the 30-days follow-up of the prehospital HEART score using serial hs-cTnT levels

| Clinical endpoints | Total (n = 304) | HEART-score ≤3 (n = 36) | HEART-score >3 (n = 268) |
| --- | --- | --- | --- |
| MACE | 43 (14.1%) | 2 (5.6%) | 41 (15.3%) |
| All-cause mortality | 2 (0.7%) | 0 (0.0%) | 2 (0.7%) |
| Cardiovascular mortality | 2 (0.7%) | 0 (0.0%) | 2 (0.7%) |
| Non-CV mortality | 0 (0.0%) | 0 (0.0%) | 0 (0.0%) |
| ACS | 41 (13.5%) | 2 (5.6%) | 39 (14.6%) |
| STEMI | 7 (2.3%) | 0 (0.0%) | 7 (2.6%) |
| NSTEMI | 34 (11.2%) | 2 (5.6%) | 32 (11.9%) |
| Unstable angina pectoris | 0 (0.0%) | 0 (0.0%) | 0 (0.0%) |
| Type 2 MI | 0 (0.0%) | 0 (0.0%) | 0 (0.0%) |
| PCI | 5 (1.6%) | 0 (0.0%) | 5 (1.9%) |
| Urgent PCI | 2 (0.7%) | 0 (0.0%) | 2 (0.7%) |
| CABG | 1 (0.3%) | 0 (0.0%) | 1 (0.4%) |
| Urgent CABG | 1 (0.3%) | 0 (0.0%) | 1 (0.4%) |
| CVA | 1 (0.3%) | 0 (0.0%) | 1 (0.4%) |
| TIA | 0 (0.0%) | 0 (0.0%) | 0 (0.0%) |
| Readmission | 19 (6.3%) | 2 (5.6%) | 17 (6.3%) |
| Cardiac readmission | 14 (4.6%) | 1 (2.8%) | 13 (4.9%) |
| Non-cardiac readmission | 5 (1.6%) | 1 (2.8%) | 4 (1.5%) |

*MACE = Cardiovascular mortality, myocardial infarction (STEMI/NSTEMI), urgent revascularization at 30 days; ACS = Acute Coronary Syndrome, including STEMI/NSTEMI/UAP; PCI = Percutaneous Coronary Intervention; CABG = Coronary Artery Bypass Grafting; CVA/TIA = Cerebrovascular accident or transient ischemic attack*

**Supplementary Table 11.** Primary endpoints in the total population using the prehospital HEART score (serial hs-cTnI levels at T0 and T1)

| Clinical endpoints | Total (n = 223) | HEART-score ≤3 (n = 55) | HEART-score >3 (n = 168) |
| --- | --- | --- | --- |
| MACE | 31 (13.9%) | 3 (5.5%) | 28 (16.7%) |
| All-cause mortality | 2 (0.9%) | 0 (0.0%) | 2 (1.2%) |
| Cardiovascular mortality | 2 (0.9%) | 0 (0.0%) | 2 (1.2%) |
| Non-CV mortality | 0 (0.0%) | 0 (0.0%) | 0 (0.0%) |
| ACS | 29 (13.0%) | 3 (5.5%) | 26 (15.5%) |
| STEMI | 4 (1.8%) | 0 (0.0%) | 4 (2.4%) |
| NSTEMI | 26 (11.7%) | 3 (5.5%) | 23 (13.7%) |
| Unstable angina pectoris | 0 (0.0%) | 0 (0.0%) | 0 (0.0%) |
| Type 2 MI | 0 (0.0%) | 0 (0.0%) | 0 (0.0%) |
| PCI | 4 (1.8%) | 0 (0.0%) | 4 (2.4%) |
| Urgent PCI | 2 (0.9%) | 0 (0.0%) | 2 (1.2%) |
| CABG | 1 (0.4%) | 0 (0.0%) | 1 (0.6%) |
| Urgent CABG | 1 (0.4%) | 0 (0.0%) | 1 (0.6%) |
| CVA | 1 (0.4%) | 0 (0.0%) | 1 (0.6%) |
| TIA | 0 (0.0%) | 0 (0.0%) | 0 (0.0%) |
| Readmission | 13 (5.8%) | 2 (3.6%) | 11 (6.5%) |
| Cardiac readmission | 9 (4.0%) | 1 (1.8%) | 8 (4.8%) |
| Non-cardiac readmission | 4 (1.8%) | 1 (1.8%) | 3 (1.8%) |

*MACE = Cardiovascular mortality, myocardial infarction (STEMI/NSTEMI), urgent revascularization at 30 days; ACS = Acute Coronary Syndrome, including STEMI/NSTEMI/UAP; PCI = Percutaneous Coronary Intervention; CABG = Coronary Artery Bypass Grafting; CVA/TIA = Cerebrovascular accident or transient ischemic attack.*

**Supplementary Table 12.** Primary endpoints in patients with symptoms longer than 2 hours.

| Clinical endpoints | Total (n = 193) | HEART-score ≤3 (n = 46) | HEART-score >3 (n = 147) |
| --- | --- | --- | --- |
| MACE | 27 (14.0%) | 2 (4.3%) | 25 (17.0%) |
| All-cause mortality | 2 (1.0%) | 0 (0.0%) | 2 (1.4%) |
| Cardiovascular mortality | 2 (1.0%) | 0 (0.0%) | 2 (1.4%) |
| Non-CV mortality | 0 (0.0%) | 0 (0.0%) | 0 (0.0%) |
| ACS | 39 (20.2%) | 2 (4.3%) | 37 (25.2%) |
| STEMI | 3 (1.6%) | 0 (0.0%) | 3 (2.0%) |
| NSTEMI | 22 (11.4%) | 2 (4.3%) | 20 (13.6%) |
| Unstable angina pectoris | 12 (6.2%) | 0 (0.0%) | 12 (8.2%) |
| Type 2 MI | 2 (1.0%) | 0 (0.0%) | 2 (1.4%) |
| Other cardiac condition (non-ACS) | 30 (15.5%) | 5 (10.9%) | 25 (17.0%) |
| PCI | 1 (0.5%) | 0 (0.0%) | 1 (0.7%) |
| Urgent PCI | 0 (0.0%) | 0 (0.0%) | 0 (0.0%) |
| CABG | 1 (0.5%) | 0 (0.0%) | 1 (0.7%) |
| Urgent CABG | 1 (0.5%) | 0 (0.0%) | 1 (0.7%) |
| CVA | 1 (0.5%) | 0 (0.0%) | 1 (0.7%) |
| TIA | 0 (0.0%) | 0 (0.0%) | 0 (0.0%) |
| Readmission | 10 (5.2%) | 2 (4.3%) | 8 (5.4%) |
| Cardiac readmission | 7 (3.6%) | 0 (0.0%) | 7 (4.8%) |
| Non-cardiac readmission | 3 (1.6%) | 2 (4.3%) | 1 (0.7%) |

**Supplementary Table 13.** Primary endpoints in patients with symptom duration ≥2h using pre-hospital HEART-score (serial hs-cTnT at T0 and T1)

| Clinical endpoints | Total (n = 179) | HEART-score ≤3 (n = 23) | HEART-score >3 (n = 156) |
| --- | --- | --- | --- |
| MACE | 28 (15.6%) | 1 (4.3%) | 27 (17.3%) |
| All-cause mortality | 2 (1.1%) | 0 (0.0%) | 2 (1.3%) |
| Cardiovascular mortality | 2 (1.1%) | 0 (0.0%) | 2 (1.3%) |
| Non-CV mortality | 0 (0.0%) | 0 (0.0%) | 0 (0.0%) |
| ACS | 26 (14.5%) | 1 (4.3%) | 25 (16.0%) |
| STEMI | 3 (1.7%) | 0 (0.0%) | 3 (1.9%) |
| NSTEMI | 22 (12.3%) | 1 (4.3%) | 21 (13.5%) |
| Unstable angina pectoris | 0 (0.0%) | 0 (0.0%) | 0 (0.0%) |
| Type 2 MI | 0 (0.0%) | 0 (0.0%) | 0 (0.0%) |
| PCI | 3 (1.7%) | 0 (0.0%) | 3 (1.9%) |
| Urgent PCI | 0 (0.0%) | 0 (0.0%) | 0 (0.0%) |
| CABG | 1 (0.6%) | 0 (0.0%) | 1 (0.6%) |
| Urgent CABG | 1 (0.6%) | 0 (0.0%) | 1 (0.6%) |
| CVA | 1 (0.6%) | 0 (0.0%) | 1 (0.6%) |
| TIA | 0 (0.0%) | 0 (0.0%) | 0 (0.0%) |
| Readmission | 9 (5.0%) | 1 (4.3%) | 8 (5.1%) |
| Cardiac readmission | 7 (3.9%) | 0 (0.0%) | 7 (4.5%) |
| Non-cardiac readmission | 2 (1.1%) | 1 (4.3%) | 1 (0.6%) |

**Supplementary Table 14.** Primary endpoints in patients with symptom duration ≥2h using pre-hospital HEART-score (serial hs-cTnI at T0 and T1)

| Clinical endpoints | Total (n = 127) | HEART-score ≤3 (n = 30) | HEART-score >3 (n = 97) |
| --- | --- | --- | --- |
| MACE | 22 (17.3%) | 1 (3.3%) | 21 (21.6%) |
| All-cause mortality | 2 (1.6%) | 0 (0.0%) | 2 (2.1%) |
| Cardiovascular mortality | 2 (1.6%) | 0 (0.0%) | 2 (2.1%) |
| Non-CV mortality | 0 (0.0%) | 0 (0.0%) | 0 (0.0%) |
| ACS | 20 (15.7%) | 1 (3.3%) | 19 (19.6%) |
| STEMI | 2 (1.6%) | 0 (0.0%) | 2 (2.1%) |
| NSTEMI | 18 (14.2%) | 1 (3.3%) | 17 (17.5%) |
| Unstable angina pectoris | 0 (0.0%) | 0 (0.0%) | 0 (0.0%) |
| Type 2 MI | 0 (0.0%) | 0 (0.0%) | 0 (0.0%) |
| PCI | 2 (1.6%) | 0 (0.0%) | 2 (2.1%) |
| Urgent PCI | 0 (0.0%) | 0 (0.0%) | 0 (0.0%) |
| CABG | 1 (0.8%) | 0 (0.0%) | 1 (1.0%) |
| Urgent CABG | 1 (0.8%) | 0 (0.0%) | 1 (1.0%) |
| CVA | 1 (0.8%) | 0 (0.0%) | 1 (1.0%) |
| TIA | 0 (0.0%) | 0 (0.0%) | 0 (0.0%) |
| Readmission | 7 (5.5%) | 1 (3.3%) | 6 (6.2%) |
| Cardiac readmission | 5 (3.9%) | 0 (0.0%) | 5 (5.2%) |
| Non-cardiac readmission | 2 (1.6%) | 1 (3.3%) | 1 (1.0%) |

**Supplementary Table 15.** Diagnostic performance of prehospital strategies for MI and MACE: sensitivity, specificity, predictive values, and AUC in patients with symptoms >2 h

| Diagnostic strategy | Sensitivity (%) | Specificity (%) | PPV (%) | NPV (%) | AUC (95% CI) |
| --- | --- | --- | --- | --- | --- |
| *MI* |  |  |  |  |  |
| Pre-hospital HEART ≤ 3 with conventional POC-cTnI | 92.6 | 26.5 | 17.0 | 95.7 | 0.687 (0.59 – 0.79)^a^ |
| Pre-hospital HEART≤ 3 with serial hs-cTnT | 95.8 | 11.6 | 37.7 | 83.3 | 0.60 (0.46–0.74)^b^ |
| Pre-hospital HEART≤ 3 with serial hs-cTnI | 95 | 27.1 | 19.6 | 96.7 | 0.78 (0.67 – 0.83)^c^ |
| In-hospital ESC 0/1-h hs-cTnT reference (T1–T2) ^f^ | 100 | 94.3 | 90.9 | 100 | 0.97 (0.91 – 1.00) |
|  |  |  |  |  |  |
| *MACE* |  |  |  |  |  |
| Pre-hospital HEART ≤ 3 with conventional POC-cTnI | 93.1 | 26.8 | 18.4 | 95.7 | 0.70 (0.61 – 0.8)^a^ |
| Pre-hospital HEART≤ 3 with serial hs-cTnT | 96.2 | 12.2 | 41 | 83.3 | 0.60 (0.47–0.74)^d^ |
| Pre-hospital HEART≤ 3 with serial hs-cTnI | 95.2 | 27.6 | 21.6 | 96.7 | 0.78 (0.68 – 0.88)^e^ |
| In-hospital ESC 0/1-h hs-cTnT reference (T1–T2) ^f^ | 100 | 94.3 | 90.9 | 100 | 0.93 (0.98 – 1.00) |

^a^ p-value <0.001

^b^ p –value 0.07

^c^ p –value 0.03

^d^ p –value 0.06

^e^ p-value 0.02

^f^ golden standard

POC, point of care; cTnI, cardiac troponin I; hs-cTnI, high-sensitive cardiac troponin I; hs-cTnT, high-sensitive cardiac troponin T;

**Supplementary box 1. Additional methodological details**

**Study design**

In addition to a primary comparison using a conventional POC cTnI assay, we performed exploratory analyses by incorporating central laboratory-based serial hs-cTnT and hs-cTnI measurements into the HEART score.

**Study population**The exclusion criteria were cognitive impairment, pregnancy, haemodynamic instability or shock, neurological unresponsiveness, unavailability of 12-lead prehospital ECG, ST-segment elevation on ECG (compatible with STEMI), obvious noncardiac chest pain (e.g., trauma), or suspicion of aortic dissection or pulmonary embolism. The study was terminated prematurely after 400 inclusions instead of the planned 650 and was formally concluded on 1 August 2023 for the following reasons: (i) availability of POC hs-cTn assays, making the studied conventional POC cTnI assay less relevant; (ii) limited availability of POC devices, logistical constraints, and the impact of the COVID-19 pandemic on enrolment; and (iii) exhaustion of the allocated budget, which was originally intended for 6–12 months of inclusion.

**Study protocol**

Although the i-STAT device has been formally validated only for use with venous whole blood or plasma, capillary sampling was used in this study for practical and logistic reasons in a pre-hospital setting. Prior to study roll-out, local verification of capillary whole blood use was performed. Nevertheless, sampling-related variability may affect measurements near decision limits and should be considered when interpreting our findings.[16] Venous blood was obtained for subsequent high-sensitivity troponin analysis.

**Troponin testing and cutoff values**

Prehospital capillary POC cTnI levels were measured using an i-STAT 1 analyser (Abbott Point of Care Inc., Princeton, NJ, USA). In-hospital hs-cTnT was measured using the Elecsys® hs-cTnT assay on the cobas® platform (Roche Diagnostics, Mannheim, Germany). Retrospective hs-cTnI values were obtained from stored plasma using the Beckman Coulter Access hs-cTnI assay.

**Exploratory sub-analyses**

In addition to the primary comparison, we performed the following exploratory sub-analyses. An alternate pre-hospital HEART score was constructed by incorporating serial hs-cTnT and hs-cTnI values at T0 (pre-hospital) and T1 (first in-hospital medical contact). Troponin scoring followed the ESC 0/1-h algorithm, assigning 0, 1, or 2 points to the troponin component based on the rule-out, observation, or rule-in thresholds. We also evaluated a combined pre-hospital HEAR ≤3 and POC conventional cTnI score = 0 strategy. As a stricter risk classification, both a HEART score ≤3 and a troponin sub-score of 0 were required. The reference standard for all comparisons was the in-hospital serial hs-cTnT test result (T1 and, when clinically indicated, T2), interpreted according to the ESC of Cardiology 0/1-h algorithm to confirm or exclude myocardial injury based on dynamic troponin changes.

**Primary endpoints**

The primary endpoints included interobserver agreement of pre- and in-hospital HEART scores, measured using intraclass correlation coefficients (ICCs), and the diagnostic performance of different HEART score strategies for ruling out myocardial infarction (MI) and major adverse cardiac events (MACE). MI was defined according to the Fourth Universal Definition of MI, requiring a rise and/or fall in cardiac troponin levels with at least one value above the 99th percentile upper reference limit in combination with clinical evidence of myocardial ischaemia, such as symptoms, ECG changes, or imaging abnormalities. MACE was defined as a composite of cardiovascular mortality, MI, and urgent coronary revascularisation, including non-elective percutaneous coronary intervention (PCI) or coronary artery bypass grafting (CABG) within 30 days after the first medical contact.

**Statistical analysis**

Receiver operating characteristic curves were constructed to calculate the area under the curve (AUC) for the discrimination analysis. Missing data patterns were examined using Little’s missing completely random (MCAR) test. Because the MCAR assumption was not violated, a complete case analysis was conducted. Continuous variables were presented as means (±SD) or medians (interquartile range), depending on normality. Categorical variables were expressed as proportions. Group comparisons were performed using Fisher’s exact test, paired t-test, or Wilcoxon signed-rank test, as appropriate.

To evaluate the ESC 0/1-h algorithm, the patients were categorised as rule-out, observe, or rule-in. The diagnostic metrics (sensitivity, specificity, NPV, and PPV) were calculated for the rule-out group. 'Observe' patients were grouped with non-rule-out cases.

**Power calculation**

The evaluation of the pre-hospital triage pathways for ruling out MI and MACE at 30 days required approximately 110 participants, assuming a 15% event rate, to achieve reliable 95% confidence intervals around a target sensitivity of 99%; this requirement was met as the cohort comprised 331 patients, including 46 with index NSTEMIs and 46 with MACE (13.9%),

A non-inferiority comparison of serial POC-cTnI sampling (T0–T1) with laboratory hs-cTn assays was originally planned, but was later withdrawn. Recruitment was halted before the anticipated 650 participants could be enrolled, and the validated 0/1-h cutoff values for the POC conventional cTnI assay were unavailable. Follow-up was nearly complete, with missing outcome data for only four patients (overall attrition, 1%), well below the 10% allowance in the original protocol.

**Supplementary Box 2. The HEART score variables**
The HEART score assigns points (0–10) across five components: history, ECG, age, cardiovascular risk factors, and troponin. Each component was scored 0–2 points, and the patients were categorised into low-(0–3 points), moderate-(4–6 points), or high-risk (7–10 points) groups.

| Component | 0 points | 1 point | 2 points |
| --- | --- | --- | --- |
| History | Slightly suspicious | Moderately suspicious | Highly suspicious of ischemia |
| ECG | Normal | Non-specific repolarization disturbance | Significant ST-deviation suggestive of ischemia |
| Age | <45 years | 45–64 years | ≥65 years |
| Risk factors | No known cardiovascular risks | 1–2 risk factors | ≥3 risk factors or known atherosclerotic disease |
| Troponin | Normal | Elevated <3× normal limit | Elevated ≥3× normal limit |

**Supplementary Box 3. Troponin cutoff values**

Troponin cutoff values used according to manufacturer guidelines and ESC recommendations^1^:

| Assay | Rule-out | Rule-in |
| --- | --- | --- |
| POC Troponin I (i-Stat, Abbott) | <0.02 µg/L | ≥0.08 µg/L |
| hs-cTnT (Roche) | <0.005 µg/L | ≥0.052 µg/L |
| Hs-cTnI (Beckman) | ≤0.004 µg/L | ≥0.023 µg/L |

**Supplementary Box 4. Study results**

**Study population**

In 52 patients, the HEART score could not be calculated, mainly because the prehospital POC cTnI test failed (n=26) or because one of the HEART components had not been documented (n=26).

**Baseline characteristics**

The mean age of the stratified groups was significantly lower in the low-risk group than in the intermediate-to high-risk group (56.1 vs. 68.7 years, p<0.001). The low-risk group had more female patients (61.6% vs. 43.3%, p=0.005) and lower prevalence rates of diabetes mellitus (12.8% vs. 25.3%, p=0.024), hypercholesterolaemia (30.2% vs. 61.1%, p<0.001), and hypertension (24.4% vs. 65.7%, p<0.001). Cardiovascular history was significantly less common among low-risk patients with fewer previous MIs (4.7% vs. 36.2%, p<0.001), PCI (4.7% vs. 32.1%, p<0.001), and no prior CABG than in the intermediate-to-high-risk group (12.3% vs. 12.3 %, p=0.001). A complete overview of the baseline characteristics is provided in Table 1.

**Agreement pre-hospital and in-hospital HEART-score**

The agreement was the highest for age (ICC=0.905; 94.9%) and lowest for history (0.212; 42.9%) and ECG (0.387; 67.1%), with intermediate values for risk factors (0.542; 62.2%) and troponin (0.460; 69.8%).

The prehospital and in-hospital HEART score classifications differed in 23% of the patients (75/331, Supplementary Table 4), mainly due to subjective components such as history and risk factors (Supplementary Table 5). Of the 20 patients classified as low-risk in the prehospital setting but high-risk in the hospital setting (false negatives), two experienced MACE (both NSTEMIs), while no deaths or urgent revascularization occurred (Supplementary Table 6). False positives (n=55) were explained by (ambulance) overestimation of history.

**Exploratory sub-analyses**

In addition to the primary analysis, we conducted the following analyses.

**Alternate pre-hospital HEART score incorporating serial hs-cTnT scoring (central laboratory):**

For 304 of the 331 patients (91.8%), the venous hs-cTnT levels were available both preoperatively (T0) and upon ED arrival (T1). For MI, the sensitivity reached 95.1%, specificity was 12.9%, NPV was 94.4%, and AUC was 0.70 (95% CI 0.62–0.79). For MACE, the corresponding values were 95.3%, 13.0%, 94.4%, and 0.70 (95% CI 0.62–0.79).

**Alternate pre-hospital HEART score incorporating serial hs-cTnI scoring (central laboratory):**

For 223 of 331 patients (67.4%), venous hs-cTnI values were available both at the pre-hospital stage (T0) and upon ED arrival (T1). For MI, the sensitivity was 93.5%, specificity was 26.8%, NPV was 94.5%, and AUC was 0.75 (95% CI 0.65–0.85). For MACE, the corresponding values were 90.3%, 27.1%, 94.5%, and 0.75 (95% CI 0.66–0.85).

**Combined pre-hospital HEAR ≤3 and POC conventional cTnI sub-score 0 strategy:**

On evaluating patients who had both a HEART score ≤3 and a troponin sub-score of 0 using the POC conventional cTnI assay; for MI, the sensitivity was 93.5%, specificity was 28.4%, NPV was 96.4%, and AUC was 0.39 (95% CI 0.35–0.44); for MACE, the corresponding values were 94.4%, 27.6%, 96.7%, and 0.39 (95% CI 0.34–0.44).

**Clinical outcomes in the pre-hospital low-risk group**

Using the pre-hospital HEART score incorporating serial hs-cTnT (T0 and T1), available in 304 patients, 36 individuals (11.8%) were classified as low risk (HEART ≤3), with two MACEs (5.6%) in this group—all index NSTEMIs (Supplementary Table 10). Similarly, when applying serial hs-cTnI scoring in 223 patients, 55 (24.7%) were categorised as low risk, with three MACEs (5.5%) observed (Supplementary Table 11). These findings show that the use of serial hs-cTnT or hs-cTnI reduced the number of missed MACEs from four (4.7%) in the original POC-cTnI strategy to two or three events, while maintaining a comparable NPV.

**Influence of symptom duration**

When the alternate pre-hospital HEART score with serial hs-cTnT was applied in this ≥2-h population (n=179 patients with both hs-cTnT values and complete HEART-scores at T0 and T1), 23 patients (12.8%) were classified as low risk, of whom one experienced a MACE (4.3%; Supplementary Table 13). Similarly, when serial hs-cTnI values were used in the same sub-group (n = 127), 30 patients (23.6%) were classified as low risk, with one MACE (3.3%) observed (Supplementary Table 14). Thus, the application of this strategy in a symptom duration-defined population further reduced the number of missed MACEs in the low-risk group from two to one, while maintaining a comparable proportion of low-risk patients (12.8% vs. 11.8% in the total cohort). An overview of the diagnostic performance of the pre-hospital and alternate pre-hospital HEART-score strategies in this ≥2-h symptom-duration sub-group is provided in Supplementary Table 15.

**References are described in the main paper.**
